# Next-generation phenotyping: introducing phecodeX for enhanced discovery research in medical phenomics

**DOI:** 10.1093/bioinformatics/btad655

**Published:** 2023-11-01

**Authors:** Megan M Shuey, William W Stead, Ida Aka, April L Barnado, Julie A Bastarache, Elly Brokamp, Meredith Campbell, Robert J Carroll, Jeffrey A Goldstein, Adam Lewis, Beth A Malow, Jonathan D Mosley, Travis Osterman, Dolly A Padovani-Claudio, Andrea Ramirez, Dan M Roden, Bryce A Schuler, Edward Siew, Jennifer Sucre, Isaac Thomsen, Rory J Tinker, Sara Van Driest, Colin Walsh, Jeremy L Warner, Quinn S Wells, Lee Wheless, Lisa Bastarache

**Affiliations:** Department of Medicine, Vanderbilt University Medical Center, Nashville, TN 37203, United States; Vanderbilt Genetics Institute, Vanderbilt University Medical Center, Nashville, TN 37203, United States; Department of Biomedical Informatics, Vanderbilt University Medical Center, Nashville, TN 37203, United States; Department of Biomedical Informatics, Vanderbilt University Medical Center, Nashville, TN 37203, United States; Department of Biomedical Informatics, Vanderbilt University Medical Center, Nashville, TN 37203, United States; Department of Medicine, Vanderbilt University Medical Center, Nashville, TN 37203, United States; Vanderbilt Genetics Institute, Vanderbilt University Medical Center, Nashville, TN 37203, United States; Department of Pediatrics, Virginia Commonwealth University, Richmond, VA 23219, United States; Department of Biomedical Informatics, Vanderbilt University Medical Center, Nashville, TN 37203, United States; Department of Pathology, Northwestern Feinberg School of Medicine, Chicago, IL 60611, United States; Department of Biomedical Informatics, Vanderbilt University Medical Center, Nashville, TN 37203, United States; Department of Medicine, Vanderbilt University Medical Center, Nashville, TN 37203, United States; Department of Medicine, Vanderbilt University Medical Center, Nashville, TN 37203, United States; Vanderbilt Genetics Institute, Vanderbilt University Medical Center, Nashville, TN 37203, United States; Department of Biomedical Informatics, Vanderbilt University Medical Center, Nashville, TN 37203, United States; Department of Ophthalmology, Vanderbilt University Medical Center, Nashville, TN 37232, United States; All of Us Research Program, National Institutes of Health, Bethesda, MD 20892, United States; Department of Medicine, Vanderbilt University Medical Center, Nashville, TN 37203, United States; Vanderbilt Genetics Institute, Vanderbilt University Medical Center, Nashville, TN 37203, United States; Department of Pediatrics, Vanderbilt University Medical Center, Nashville, TN 37203, United States; Department of Medicine, Vanderbilt University Medical Center, Nashville, TN 37203, United States; Department of Pediatrics, Vanderbilt University Medical Center, Nashville, TN 37203, United States; Department of Pediatrics, Vanderbilt University Medical Center, Nashville, TN 37203, United States; Department of Pediatrics, Vanderbilt University Medical Center, Nashville, TN 37203, United States; All of Us Research Program, National Institutes of Health, Bethesda, MD 20892, United States; Department of Pediatrics, Vanderbilt University Medical Center, Nashville, TN 37203, United States; Department of Biomedical Informatics, Vanderbilt University Medical Center, Nashville, TN 37203, United States; Department of Pediatrics, Vanderbilt University Medical Center, Nashville, TN 37203, United States; Department of Medicine, Vanderbilt University Medical Center, Nashville, TN 37203, United States; Department of Medicine, Vanderbilt University Medical Center, Nashville, TN 37203, United States; Department of Biomedical Informatics, Vanderbilt University Medical Center, Nashville, TN 37203, United States

## Abstract

**Motivation:**

Phecodes are widely used and easily adapted phenotypes based on International Classification of Diseases codes. The current version of phecodes (v1.2) was designed primarily to study common/complex diseases diagnosed in adults; however, there are numerous limitations in the codes and their structure.

**Results:**

Here, we present phecodeX, an expanded version of phecodes with a revised structure and 1,761 new codes. PhecodeX adds granularity to phenotypes in key disease domains that are under-represented in the current phecode structure—including infectious disease, pregnancy, congenital anomalies, and neonatology—and is a more robust representation of the medical phenome for global use in discovery research.

**Availability and implementation:**

phecodeX is available at https://github.com/PheWAS/phecodeX.

## 1 Introduction

Phecodes are manually curated groups of International Classification of Diseases (ICD) codes intended to capture clinically meaningful concepts for research (Denny *et al.* 2010, Bastarache 2021). Although initially created to conduct phenome-wide association studies (PheWAS) of common genetic loci, the applications of phecodes have broadened considerably in recent years (Denny *et al.* 2010, Bastarache 2021, Bastarache *et al.* 2022). Phecodes have been used in conjunction with various methods—including machine learning, comorbidity clustering, and prediction algorithms—and to conduct research across a wide array of clinical domains, from Mendelian disease to pharmacology (Pruett *et al.* 2021, Zhang *et al.* 2021, Bastarache *et al.* 2022, McArthur *et al.* 2023, Hellwege *et al.* 2023).

Because they are based on ICD codes—a global standard for classifying disease—phecodes can be used in virtually every electronic health record-linked biobank, both in the USA and internationally (Zhou *et al.* 2022). The wide-spread use of phecodes attests to their value, however, the structure has not been revised since 2013, and the current version (v1.2) has limitations. Phecode v1.2 focused on capturing diseases present in the genome-wide association (GWAS) catalog, which was comprised mainly of common diseases of adulthood (Denny *et al.* 2013, Sollis *et al.* 2023). Phecodes relating to pregnancy, congenital anomalies, and neonatology are present in v1.2, but only as highly aggregated concepts. Furthermore, v1.2 was designed using the outdated ICD-9 coding system and does not take advantage of the greater granularity and modernized organization of ICD-10s. Here, we present phecodeX, an expanded and updated version of phecodes designed to overcome many of these limitations and further facilitate new and creative phecode applications.

The new phecodeX map differs from v1.2 in several ways. PhecodeX (1) *aligns its structure with the ICD-10 coding system*, (2) *revises the phecode labeling system*, (3) *leverages multi-mapping of both ICD-9 and -10 codes*, (4) *removes exclude ranges used to define controls*, and (5) *reorganizes phecode categories.* Because of these changes, which are detailed below, phecodeX is a more comprehensive representation of the medical phenome with enhanced coverage of phenotypes relevant to both complex and monogenic disease. We describe these differences in detail below.

## 2 Methods

PhecodeX was created via manual curation in consultation with 22 clinicians. Each expert received a draft version of phecodeX according to their expertise and provided feedback. Changes were discussed with iterative adjustments made until a final grouping was determined. This method was also employed to create v1.2, although the collective expertise was greater for phecodeX (Supplementary Table S1 describes co-author expertise). A description of the 3612 phecodes that constitute phecodeX is available in Supplementary Table S2.

PhecodeX was developed using ICD clinical modification (CM), a US extension of the ICD codes defined by the World Health Organization (WHO). ICD-10-CM shares the same structure as the WHO defined ICD-10 codes but includes more detailed codes. To support the international use of phecodes, we created a WHO ICD-10 compatible mapping file. Like phecode v1.2, the phecodeX WHO ICD map supports fewer phecodes. In the case of PhecodeX, the WHO ICD map includes 2872 of the 3612 phecodes in phecodeX. Summary information comparing phecode v1.2 and phecodeX is available in Supplementary Table S3. All phecodeX mapping files are available for download (https://github.com/PheWAS/phecodeX).

## 3 Results and discussion

### 3.1 Alignment with ICD-10 structure

**Figure 1.**
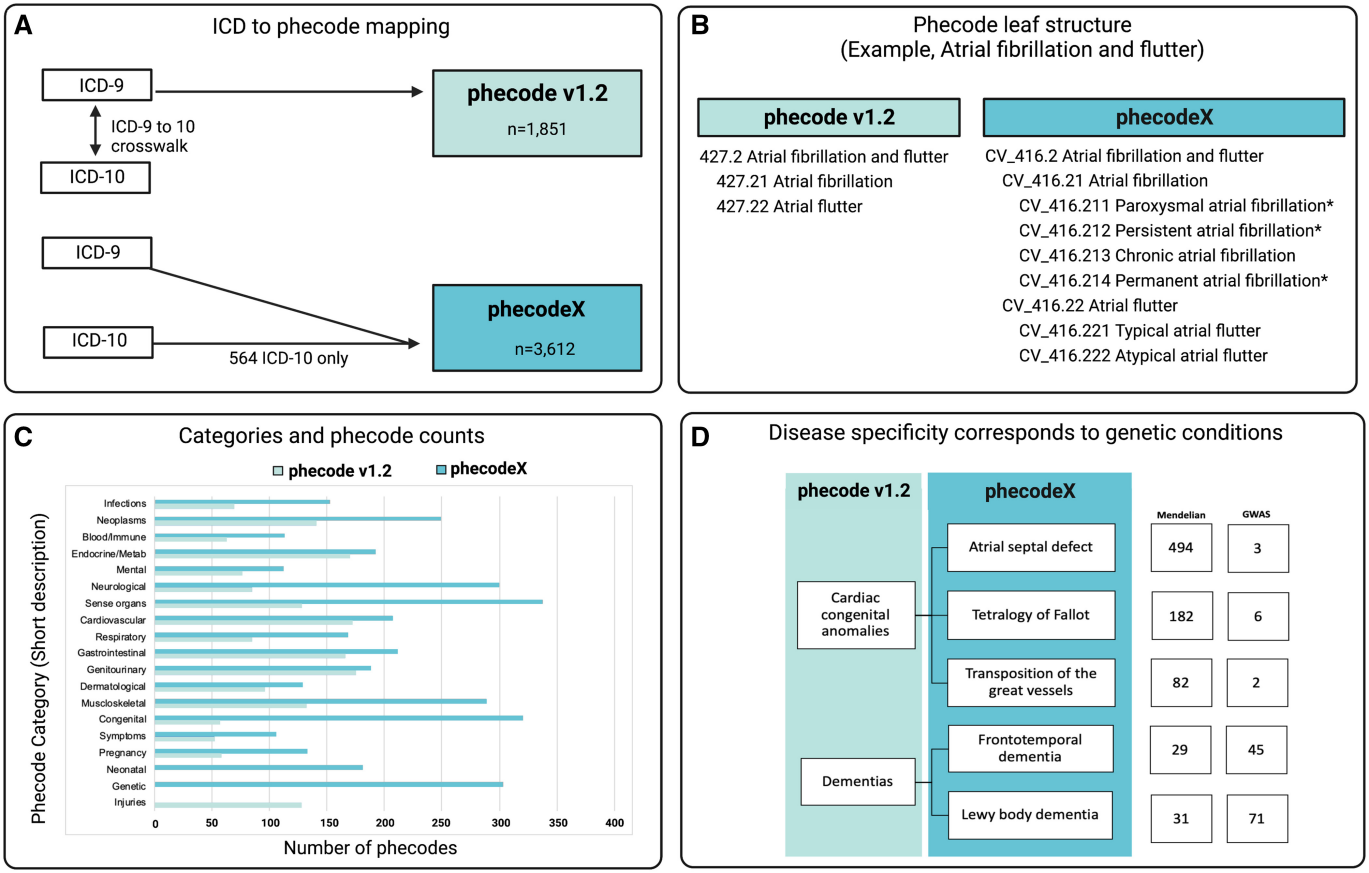
Overview of the phecodeX data structure and differences from version 1.2. Panel (A) demonstrates how phecode v1.2 was designed using codes from the ICD-9; ICD-10s were later integrated into phecodes using the ICD crosswalk. PhecodeX adapted the overall structure of ICD-10s; codes from ICD-9 and -10 were mapped simultaneously to optimize the phecode structure for both ICD versions. Panel (B) provides an example of the expansion in phecode tree structure. The new system adds a tertiary level with up to three digits past the decimal place for increased phecode specificity. This example also demonstrates the difference in phecode labels and strings, including the introduction of a two-character category descriptor (e.g. CV for the Cardiovascular category) and the use of * after code description to denote codes that map to only ICD-10 billing codes. Panel (C) demonstrates the difference in the size of phecodeX versus v1.2, stratified by phecode category. Finally, panel (D) shows how new phecodes introduced in phecodeX improve coverage of phenotypes relevant to both complex and Mendelian disease. In this example, the two phecodes in v1.2 are expanded to reflect five phecodes in phecodeX. The column labeled Mendelian indicates the number of Mendelian disease genes linked to the phenotypes through the Human Phenotype Ontology. The column labeled GWAS indicates the number of unique genetic variants present in the GWAS catalog. This figure was created with BioRender.com.

Phecode v1.2 was designed using the ICD-9-CM coding system and thus did not take advantage of the increased granularity and modernized taxonomy of ICD-10. By updating its structure to *align with the ICD-10 coding system*, phecodeX takes advantage of the increased granularity available in the ICD-10 schema. The ICD-10 coding structure includes nearly 10 times as many codes compared with ICD-9 (Steindel 2010, Fung *et al.* 2016). While ICD-10 codes have been integrated into v1.2, no new phecodes were introduced in this process (Wu *et al.* 2019). Thus, the design of v1.2 was based on ICD-9 and did not take advantage of the increased specificity of ICD-10s. PhecodeX includes 572 new phecodes (Fig. 1A) representing diagnoses that were newly added to the ICD-10-CM system (e.g. dilated cardiomyopathy, COVID-19, and gestational diabetes). Phecodes supported by ICD-10 alone are denoted in the icd10_only column of the mapping file and their phecode string ends with an *.

PhecodeX also reflects the more modern disease classification of the ICD-10 coding structure. ICD-9 codes were created and organized based on the understanding of diseases in 1977, when they were developed (Fung, *et al.* 2016). ICD-10 updated this structure by recategorizing diseases like Macroglobulinemia, which was situated in the Endocrine/Metabolic chapter, to the Neoplasms chapter. These changes in disease classification are reflected in the phecodeX coding structure.

### 3.2 New phecode labels

Each phecodeX label is prefixed by a two-letter label indicating the category, followed by an underscore and a three-digit root code. In contrast, v1.2 phecodes were labeled with three-digit root codes, similar to ICD-9s. The character prefixes make phecodeX visually distinct from v1.2 and ICD codes, and prevent programs like R and Excel from corrupting codes by interpreting them as integers (e.g. phecode 008 being transformed to 8). The numeric component of each phecodeX code is unique, even without the prefix.

PhecodeX allows for three decimals to indicate child codes, as opposed to v1.2 that allowed for a maximum of two digits. By adding a tertiary level of specificity, phecodeX includes more granular codes than were possible in v1.2. For example, atrial fibrillation and flutter form the basis for the code CV_416.2 (427.2 in v1.2) which is further delineated into two secondary child codes—fibrillation (416.21) and flutter (416.22)—which are the final specification for these conditions. In phecodeX, a tertiary child provides additional granularity reflecting greater specificity, for example, atrial flutter (CV_416.22) is further separated into typical and atypical flutter (CV_416.221 and CV_416.222, respectively) (Fig. 1B).

### 3.3 Multi-mapping

PhecodeX embraces multi-mapping where one ICD code can map to more than one phecode. Phecode v1.2 is based on a 1-to-1 mapping (each ICD-9 mapped to a unique phecode). Multi-mapping was introduced to integrate ICD-10 codes into the v1.2 structure to accommodate situations when an ICD-10 code represented multiple ICD-9 codes. However, multi-mapping was not used to define any new phecodes (Wu *et al.* 2019). The 1-to-1 constraint of v1.2 led to difficulties in classifying pre-coordinated ICD codes (i.e. codes that represent multiple concepts) (Boone 2011). Pre-coordinated codes are common in the infectious disease (ID) chapters, where a single code often represents both the diagnosis and infectious agent (e.g. *Pneumonia due to staphylococcus*; ICD-10 J15.2 and ICD-9 482.4) (Lu 2005). For example, in v1.2 the ICD for Pneumonia due to staphylococcus is mapped to 480.1 (*Bacterial pneumonia*) but not 041.1 (*Staphylococcus infections*), while in phecodeX, this ICD is mapped to both phenotypes (ID_009 and RE_468.2, respectively). Multi-mapping enabled a restructuring of the phecodeX ID category, whereby all infectious agents are organized by genus for bacteria and fungi, and family for viruses.

Multi-mapping pre-coordinated ICDs enabled a more complete representation of many concepts. For example, the ICDs for *Inguinal hernia, with gangrene* (ICD-9 550.0 and ICD-10 K40.1) are mapped to phecodes for *Inguinal hernia* (GI_520.11) and *Gangrene* (ID_091). In v1.2, they are only mapped to *Inguinal hernia* (550.1). Pregnancy-related codes are also frequently multi-mapped; the phecode for *Type 1 diabetes* (EM_202.1) includes ICDs from the endocrine/metabolic chapter (E10*), as well as perinatal codes like O24.01 (*Pre-existing type 1 diabetes mellitus, in pregnancy*). Using multi-mapping, we also created four phecodes to represent higher level disease categories like autoimmune disease which are described in Supplementary Table S4.

In v1.2, 7883 of the 82 827 ICD-10-CM codes (9.5%) map to more than one phecode, affecting 190 of 1309 leaf phecodes. In phecodeX, 6829 of 54 520 ICD-10-CM codes (12.5%) and 3507 of 12 665 ICD-9-CM codes were mapped to more than one phecode, affecting 20% (543 of 2688) of leaf codes (excluding the four high-level phecodes).

### 3.4 Removal of exclude ranges

PhecodeX does not include specific exclude ranges for the controls. Exclude ranges are lists of phecodes that are used to remove controls with potentially related conditions. Phecode v1.2 includes an exclude range for each phecode and they are implemented by default in the PheWAS R package (Carroll, *et al.* 2014). However, recent research suggests that exclude ranges do not globally improve the ability for phecodes to replicate known genetic associations (Bastarache *et al.* 2023). Due to the complexity introduced by exclude ranges, both in terms of executing and interpreting PheWAS analyses, combined with limited evidence for their benefit, we elected not to include these ranges in phecodeX.

### 3.5 phecodeX categories

Phecodes are grouped into categories, similar to ICD chapters. PhecodeX includes 18 categories that largely mirror those in v1.2 with three exceptions (Fig. 1C). First, phecodeX has a category for neonatal conditions. In v1.2, both neonatal and pregnancy-related phecodes fall under the category of pregnancy complications. Second, phecodeX eliminates the v1.2 category for injuries and poisonings, which includes 128 phecodes for traumatic injuries, poisonings, and surgical complications defined with ICDs from chapters Injuries and poisonings and External causes of morbidity. In phecodeX, codes for fractures and dislocations have been moved to the musculoskeletal category. The remaining injuries and poisonings codes are excluded from phecodeX. These codes indicate an exposure rather than a diagnosis (e.g. *retained foreign body in the eye* or *Poisoning by antibiotics*) or require a specific exposure to manifest (e.g. *intraoperative hemorrhage and hematoma of eye* or *Toxic effect of radiation*). While these codes may prove highly valuable for research, they are ill-suited to a PheWAS-style analysis which currently lacks a mechanism to define control sets on the basis of an exposure (Supplementary Table S5 lists ICD-10-CM codes that were included in phecode v1.2 but excluded from phecodeX). Finally, phecodeX introduces a new section for genetic conditions that includes 324 phecodes for diseases caused exclusively by single gene or chromosomal variants (e.g. Rett syndrome, Trisomy 18, and DiGeorge syndrome). A summary of phecodeX categories can be found in Supplementary Table S6.

### 3.6 PhecodeX and multiple testing burden

Due to these changes, phecodeX has nearly twice the number of phecodes as v1.2 (3,612 versus 1,866, respectively). PhecodeX expanded granularity is most notable in categories for pregnancy, neonatal, and congenital anomalies. PhecodeX includes 5.8 times more codes for congenital anomalies compared with v1.2 (365 versus 56) and 5.1 times more codes for pregnancy/neonatal disorders (297 versus 58).

The expansion in granularity has the benefit of a more comprehensive representation of the medical phenome, including many conditions associated with rare and common genetic drivers of disease (Fig. 1D). However, the addition of new codes can also impact multiple testing burden for analyses such as PheWAS. If all codes were included in a PheWAS, the Bonferroni corrected *P*-value for nominal significance using v1.2 would be 2.68×10^−5^ versus 1.38×10^−5^ for phecodeX. However, this difference is mitigated by the fact that many new codes in phecodeX are for rare phenotypes and diagnoses. While these codes are important for studying rare genetic diseases, many will be excluded from PheWAS analysis due to low case counts. For example, in a cohort of 93 694 individuals drawn from BioVU—Vanderbilt’s de-identified biobank—the number of phecodes with at least 100 cases (defined using minimum code count of two) was 1580 for v1.2 and 1882 for phecodeX (Bonferroni *P* of 2.70×10^−5^ versus 3.20×10^−5^).

The testing burden can further be alleviated by removing or restricting to categories relating to relevant life stages, a major improvement in the new version. For example, if a researcher is interested in identifying pregnancy-related complications phecodeX would allow for a more restrictive analysis focusing on the relevant category. This is because the newer version allows for multi-mapping that would allow for conditions relating to pregnancy, such as pregnancy-related diabetes, to map to both the endocrine and the pregnancy-related conditions categories.

### 3.7 Phecode v1.2 to phecodeX crosswalk

We created a crosswalk between v1.2 and phecodeX. In total, 1020 of the 1866 (55%) phecodes in v1.2 map to codes in phecodeX (Supplementary Table S7). There are several reasons that the remaining 45% of v1.2 codes are not present in phecodeX. First, in designing phecodeX, we avoided creating vague or non-specific phecodes by reorganizing ICDs to facilitate more specific labels. In v1.2, 213 codes (12% of all phecodes) include the string Other, NOS (not otherwise specified), or NEC (not elsewhere classified). In phecodeX, we reduced the number of non-specific phecodes 92 (2.0%) by mapping the non-specific ICD codes to the appropriate parent code. Second, because the two versions of phecodes were designed based on different ICD coding systems (ICD-9 versus ICD-10), they group multiple conditions under different parent codes. Third, 128 of the v1.2 phecodes not present in the crosswalk relate to injuries and exposure-related outcomes which, as discussed above, were not included in the phecodeX map.

Most common/complex diseases present in v1.2 are also present in phecodeX and reflect the intent of v1.2 to capture diseases present in the GWAS catalog (Denny *et al.* 2013). Of the 149 phecodes in v1.2 that map to GWAS catalog diseases, 138 (93%) are present in phecodeX. Therefore, PheWAS conducted on common genetic variants may not be significantly different between the two versions. However, analysts should be aware that phecodes represented in both versions may have underlying differences in terms of their ICD groupings and the improvements in diagnostic specificities in the latter may improve the capture of particular physiologic conditions.

### 3.8 Code availability

PhecodeX is available on GitHub (https://github.com/PheWAS/phecodeX) including mapping files to support ICD-CM and the WHO standard ICD-10. PhecodeX will be periodically updated to fix any errors. Each version of PhecodeX will be archived and receive its own version number (The version described in this article is 1.001). The GitHub repository also includes support files and example code that will facilitate the use of phecodeX with the PheWAS R package.

## 4 Limitations

PhecodeX is not without limitations. First, phecodes are manually curated in collaboration with domain experts. This process is inherently subjective and non-quantitative. Despite this limitation, phecodes have been used successfully in hundreds of research projects and, critically, have enabled robust replication of known associations across international biobanks (Zhou *et al.* 2022). Second, while multi-mapping enables a more accurate representation of phenotypes, it also introduces greater redundancy in the phecode map compared with v1.2. Thus, a PheWAS using phecodeX may reveal more associations that are driven by the same ICD code. However, redundancy has always been a feature of phecodes due to the hierarchical organization and the natural correlation of the phenome. The relative increase in redundance from v1.2 and phecodeX does not alter the basic statistics or interpretation of PheWAS. Indeed, researchers have developed methods to help identify primary versus secondary PheWAS associations (Karnes *et al.* 2017, Allaire *et al.* 2023). Third, phecodeX excludes injury and trauma phecodes that are present in v1.2. Future work may re-integrate these codes with an additional framework for selecting appropriate controls. Finally, phecodeX does not define exclude ranges for control groupings. Exclude ranges have the theoretical benefit of improving the accuracy of controls by excluding similar conditions, but this claim had not been empirically tested until a recent study that showed exclude ranges did not globally improve the replicability of known genetic associations (Bastarache *et al.* 2023). Moreover, exclude ranges can complicate the interpretation of PheWAS by inducing differential cohorts for each phenotype. Given the lack of proof for their efficacy and the complexity they added to PheWAS results, we elected to forgo exclude ranges in phecodeX. More research is needed to explore this complex topic; future work may devise a more transparent and efficacious means of defining and applying exclude ranges.

## 5 Conclusions

PhecodeX (version 1.0) represents the first major revision of phecodes since the release of v1.2 in 2013. The increased specificity of phecodeX provides more granular coverage of the medical phenome intended to support new uses for phecodes and encourages further creative applications. Indeed, an alpha version of phecodeX has already been used in numerous publications, including studies on pregnancy and neonatal outcomes and hereditary cancer syndromes—studies that would not have been feasible without the additional granularity offered in phecodeX (Zeng *et al.* 2022, Campbell *et al.* 2023, Stead *et al.* 2023). We acknowledge that v1.2 remains a viable mapping for PheWAS. Indeed, many existing catalogs use v1.2; therefore, researchers may want to continue to use v1.2 for cross-site meta-analyses or replications until these resources adopt phecodeX (Zawistowski *et al.* 2023). Due to the broader and more in-depth coverage of the clinical ICD-based phenome and early adaptation of the method, we anticipate phecodeX will be a broadly applied resource for medical informatics. Future iterations of phecodeX may integrate suggestions from the broader research community to enhance specificity and applicability.

## Supplementary Material

btad655_Supplementary_Data
